# (1R*,3′S*,4′R*)-4′-(4-Chloro­phen­yl)-3′-[(4-hy­droxy-2-oxo-1,2-dihydro­quinolin-3-yl)carbon­yl]-1′-methyl­spiro­[ace­naphthyl­ene-1,2′-pyrrolidin]-2-one

**DOI:** 10.1107/S1600536811048896

**Published:** 2011-11-19

**Authors:** K. N. Vennila, M. Sankaran, P. S. Mohan, D. Velmurugan

**Affiliations:** a Centre of Advanced Study in Crystallography and Biophysics, University of Madras, Guindy Campus, Chennai 600 025, India; b School of Chemical Sciences, Bharathiar University, Coimbatore 641 046, India

## Abstract

The title compound, C_32_H_23_ClN_2_O_4_, has a quinoline, a chloro­phenyl and an acenaphthalene ring system attached to a central pyrrolidine ring, which has three stereogenic centers. Nevertheless, the compound crystallizes as a racemate with two mol­ecules of identical chirality in the asymmetric unit. They differ in the conformation of the five-membered pyrrolidine ring; in one molecule it has an envelope conformation, while in the other molecule it has a twisted conformation. In each molecule there is an intra­molecular O—H⋯O hydrogen bond making an *S*(6) ring motif. In the crystal, pairs of N—H⋯O hydrogen bonds produce inversion dimers with *R*_2_^2^(8) motifs. There are also C—H⋯O interactions present. The crystal structure contains voids (60 Å^3^) within which there is no evidence of solvent mol­ecules.

## Related literature

For the synthesis of the title compound, see: Suresh Babu *et al.* (2006 ▶); Amal Raj & Raghunathan (2003) ▶; Ponnusamy *et al.* (2007 ▶). For related structures, see: Thenmozhi *et al.* (2011 ▶); Augustine *et al.* (2010 ▶). For puckering parameters, see: Cremer & Pople (1975 ▶). For asymmetry analysis, see: Nardelli *et al.* (1983 ▶).
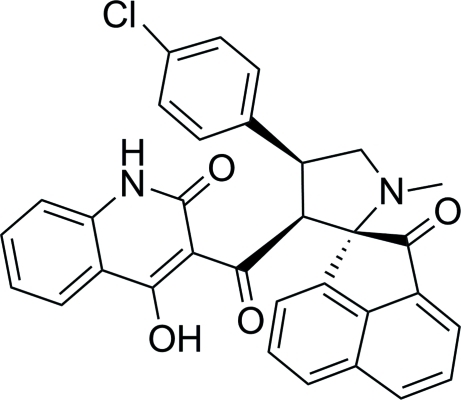


## Experimental

### 

#### Crystal data

C_32_H_23_ClN_2_O_4_
*M* *_r_* = 534.97
Triclinic, 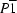

*a* = 10.7447 (7) Å
*b* = 14.1678 (9) Å
*c* = 18.3858 (11) Å
α = 101.328 (3)°
β = 91.945 (3)°
γ = 105.388 (3)°
*V* = 2635.0 (3) Å^3^
*Z* = 4
Mo *K*α radiation
μ = 0.19 mm^−1^
*T* = 293 K
0.25 × 0.24 × 0.21 mm

#### Data collection

Bruker SMART APEXII area-detector diffractometer
Absorption correction: multi-scan (*SADABS*; Bruker, 2008 ▶) *T*_min_ = 0.954, *T*_max_ = 0.961
47617 measured reflections
13107 independent reflections
6910 reflections with *I* > 2σ(*I*)
*R*_int_ = 0.029

#### Refinement

*R*[*F* ^2^ > 2σ(*F* ^2^)] = 0.047
*wR*(*F* ^2^) = 0.151
*S* = 0.94
13107 reflections
705 parameters
H-atom parameters constrained
Δρ_max_ = 0.34 e Å^−3^
Δρ_min_ = −0.42 e Å^−3^

### 

Data collection: *APEX2* (Bruker, 2008 ▶); cell refinement: *SAINT* (Bruker, 2008 ▶); data reduction: *SAINT*; program(s) used to solve structure: *SHELXS97* (Sheldrick, 2008 ▶); program(s) used to refine structure: *SHELXL97* (Sheldrick, 2008 ▶); molecular graphics: *ORTEP-3* (Farrugia, 1997 ▶); software used to prepare material for publication: *PLATON* (Spek, 2009 ▶).

## Supplementary Material

Crystal structure: contains datablock(s) global, I. DOI: 10.1107/S1600536811048896/im2325sup1.cif

Structure factors: contains datablock(s) I. DOI: 10.1107/S1600536811048896/im2325Isup2.hkl

Supplementary material file. DOI: 10.1107/S1600536811048896/im2325Isup3.cml

Additional supplementary materials:  crystallographic information; 3D view; checkCIF report

## Figures and Tables

**Table 1:** Hydrogen-bond geometry (Å, °)

*D*—H⋯*A*	*D*—H	H⋯*A*	*D*⋯*A*	*D*—H⋯*A*
N1—H1⋯O2^i^	0.86	2.03	2.879 (2)	171
O1—H1*B*⋯O3	0.82	1.74	2.468 (2)	147
N1*A*—H1*A*⋯O2*A*^ii^	0.86	2.01	2.865 (2)	174
O1*A*—H1′⋯O3*A*	0.82	1.75	2.477 (2)	148
C3*A*—H3*A*⋯O4^iii^	0.93	2.45	3.245 (3)	143
C30*A*—H30*A*⋯O2^iv^	0.93	2.54	3.413 (2)	157

Symmetry codes: (i) 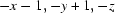; (ii) 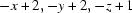; (iii) 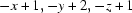; (iv) 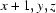.

**Fig. 1.**
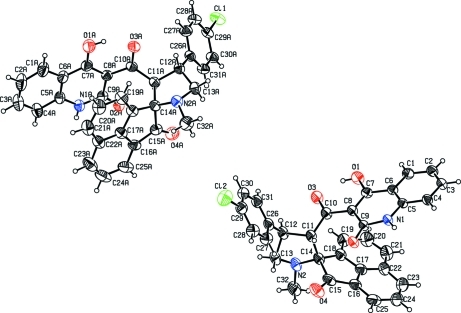
View of the title molecule showing thermal ellipsoids at the 50% probability level.

**Fig. 2.**
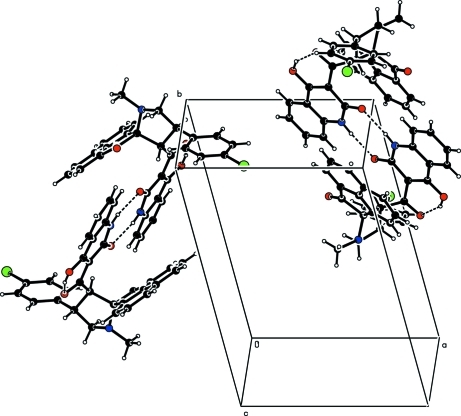
View of the motifs with hydrogen bonds (dotted lines) formed between the molecules.

## supplementary crystallographic information

### Comment

X-ray analysis confirms the molecular structure and atom connectivity of the title compound as shown in Figure 1. The pyrrolidine ring system makes dihedral angles of 90.93 (6), 94.08 (6) and 91.15 (6) ° with quinoline, acenaphthalene and chlorophenyl rings, respectively. The refined structure was observed with total potential solvent area volume of 60 Å^3^ although no solvent molecule can be detected therein.

The five membered pyrrolidine ring (C11-C13, N2, C14) adopts an envelope conformation with a two fold symmtery axis passing through C13, with the puckering parameters q2 and φ (Cremer & Pople, 1975) and the smallest displacement asymmetric parameters, Δ (Nardelli *et al.*, 1983), as follows: q2= 0.64 (3) Å, φ = 154.8 (2)°, Δs(C13) = 0.504 (2)°. The same ring is slightly twisted with regard to the N2A-C13A bond in the second molecule in the asymmetric unit. The puckering parameters q2 and φ (Cremer & Pople, 1975) and the smallest displacement asymmetric parameters, Δ (Nardelli *et al.*, 1983) of the pyrrolidine ring in the second molecule are q2= 0.67 (3) Å, φ = 151.5 (2)°, Δs(N2A) = 0.067 (1) and Δs(C13A) = 0.074 (1). The sum of angles at N2 of the pyrrolidine ring (340°) is in accordance with sp^3^ hybridization. A weak C32-H32F···cg17 interaction with a distance of 3.545 (3) Å is also observed (cg17 is the centroid of the C16, C17, C22-C25 ring).

Hydrogen bonds (Table 1) forming a R_2_^2^(8) motif link symmetry related molecule as dimers (shown with dotted lines in Figure 2).

### Experimental

A mixture of (E)-3-(3-(4-chlorophenyl)acryloyl)-4-hydroxyquinolin-2(1H)-one (0.5 mmol) , acenaphthene quinone (0.5 mmol) and sarcosine (0.55 mmol) was refluxed in methanol until the disappearance of the starting materials as evidenced by TLC. After completion of the reaction, the solvent was removed in vacuo and the residue was chromatographed on silica gel using hexane-ethylacetate mixture (7:2) as eluent to give the title compound (yield: 58%). The compound was recrystallised from a DMF-methanol mixture (2:8 v/v).

### Refinement

H-atoms were positioned geometrically and treated as riding atoms: C—H =0.93 Å H-aromatic, C—H = 0.96 Å H-methyl, and N—H = 0.86 Å, with *U*_iso_ = k×*U*_eq_(parent C or N-atom), where k = 1.5 for methyl H-atoms, and = 1.2 for all other H-atoms.

### Crystal data

**Table:**

C_32_H_23_ClN_2_O_4_	*V* = 2635.0 (3) Å^3^
*M**_r_* = 534.97	*Z* = 4
Triclinic, *P*1	*F*(000) = 1112
Hall symbol: -P 1	*D*_x_ = 1.349 Mg m^−^^3^
*a* = 10.7447 (7) Å	Mo *K*α radiation, λ = 0.71073 Å
*b* = 14.1678 (9) Å	θ = 1.1–28.4°
*c* = 18.3858 (11) Å	µ = 0.19 mm^−^^1^
α = 101.328 (3)°	*T* = 293 K
β = 91.945 (3)°	Block, colorless
γ = 105.388 (3)°	0.25 × 0.24 × 0.21 mm

### Data collection

**Table:**

Bruker SMART APEXII area-detector diffractometer	13107 independent reflections
Radiation source: fine-focus sealed tube	6910 reflections with *I* > 2σ(*I*)
graphite	*R*_int_ = 0.029
ω and φ scans	θ_max_ = 28.4°, θ_min_ = 1.1°
Absorption correction: multi-scan (*SADABS*; Bruker, 2008)	*h* = −14→14
*T*_min_ = 0.954, *T*_max_ = 0.961	*k* = −18→18
47617 measured reflections	*l* = −24→24

### Refinement

**Table:**

Refinement on *F*^2^	Primary atom site location: structure-invariant direct methods
Least-squares matrix: full	Secondary atom site location: difference Fourier map
*R*[*F*^2^ > 2σ(*F*^2^)] = 0.047	Hydrogen site location: inferred from neighbouring sites
*wR*(*F*^2^) = 0.151	H-atom parameters constrained
*S* = 0.94	*w* = 1/[σ^2^(*F*_o_^2^) + (0.0696*P*)^2^ + 0.5485*P*] where *P* = (*F*_o_^2^ + 2*F*_c_^2^)/3
13107 reflections	(Δ/σ)_max_ = 0.001
705 parameters	Δρ_max_ = 0.34 e Å^−^^3^
0 restraints	Δρ_min_ = −0.42 e Å^−^^3^

### Special details

**Table:**

Geometry. All s.u.'s (except the s.u. in the dihedral angle between two l.s. planes) are estimated using the full covariance matrix. The cell s.u.'s are taken into account individually in the estimation of s.u.'s in distances, angles and torsion angles; correlations between s.u.'s in cell parameters are only used when they are defined by crystal symmetry. An approximate (isotropic) treatment of cell s.u.'s is used for estimating s.u.'s involving l.s. planes.
Refinement. Refinement of F^2^ against ALL reflections. The weighted R-factor wR and goodness of fit S are based on F^2^, conventional R-factors R are based on F, with F set to zero for negative F^2^. The threshold expression of F^2^ > 2σ(F^2^) is used only for calculating R-factors(gt) etc. and is not relevant to the choice of reflections for refinement. R-factors based on F^2^ are statistically about twice as large as those based on F, and R- factors based on ALL data will be even larger.

### Fractional atomic coordinates and isotropic or equivalent isotropic displacement parameters (Å^2^)

**Table:**

	*x*	*y*	*z*	*U*_iso_*/*U*_eq_	
C32A	0.6670 (3)	0.5141 (2)	0.49180 (15)	0.0904 (7)	
H32A	0.6027	0.5419	0.4738	0.136*	
H32B	0.6859	0.5390	0.5446	0.136*	
H32C	0.6346	0.4424	0.4814	0.136*	
C32	−0.3307 (3)	1.03417 (17)	0.05072 (15)	0.0930 (8)	
H32D	−0.3804	1.0182	0.0913	0.139*	
H32E	−0.3883	1.0250	0.0072	0.139*	
H32F	−0.2789	1.1026	0.0639	0.139*	
Cl1	0.94664 (7)	0.60486 (5)	0.03472 (3)	0.0873 (2)	
O2A	0.98837 (13)	0.87799 (9)	0.44141 (7)	0.0621 (3)	
N1A	1.13187 (16)	0.96189 (11)	0.53970 (9)	0.0581 (4)	
H1A	1.0984	1.0111	0.5423	0.070*	
C26A	0.90685 (19)	0.56594 (14)	0.27100 (11)	0.0559 (5)	
C9A	1.08106 (18)	0.87937 (14)	0.48428 (10)	0.0521 (4)	
C5A	1.2322 (2)	0.97268 (15)	0.59168 (11)	0.0604 (5)	
C8A	1.14086 (18)	0.79780 (14)	0.47957 (10)	0.0557 (5)	
O1A	1.31369 (16)	0.74260 (13)	0.52380 (11)	0.1010 (6)	
H1'	1.2771	0.6936	0.4911	0.151*	
C12A	0.89913 (19)	0.55895 (14)	0.35147 (11)	0.0567 (5)	
H12A	0.9588	0.5212	0.3630	0.068*	
C17A	0.8743 (2)	0.76707 (15)	0.58762 (12)	0.0643 (5)	
C11A	0.93974 (18)	0.66263 (13)	0.40624 (10)	0.0523 (4)	
H11A	0.9196	0.7124	0.3811	0.063*	
N2A	0.78450 (17)	0.54291 (12)	0.45484 (9)	0.0647 (4)	
O3A	1.14633 (15)	0.63588 (11)	0.42387 (9)	0.0781 (4)	
C1OA	1.08172 (19)	0.69753 (15)	0.43389 (10)	0.0568 (5)	
C18A	0.9147 (2)	0.68525 (15)	0.55090 (11)	0.0612 (5)	
C29A	0.9307 (2)	0.58683 (15)	0.12507 (11)	0.0632 (5)	
C30A	0.8217 (2)	0.59724 (16)	0.15805 (11)	0.0657 (5)	
H30A	0.7555	0.6109	0.1316	0.079*	
C16A	0.7811 (2)	0.78996 (16)	0.54437 (13)	0.0677 (6)	
C6A	1.2965 (2)	0.89865 (16)	0.58606 (12)	0.0670 (5)	
O4A	0.68516 (16)	0.71479 (13)	0.41899 (10)	0.0881 (5)	
C7A	1.2507 (2)	0.81183 (16)	0.52715 (13)	0.0675 (6)	
C14A	0.85067 (19)	0.65057 (14)	0.47205 (11)	0.0567 (5)	
C31A	0.8106 (2)	0.58739 (16)	0.23095 (11)	0.0644 (5)	
H31A	0.7368	0.5954	0.2535	0.077*	
C28A	1.0268 (2)	0.56482 (17)	0.16250 (13)	0.0740 (6)	
H28A	1.1004	0.5574	0.1395	0.089*	
C13A	0.7675 (2)	0.50945 (15)	0.37431 (11)	0.0666 (5)	
H13A	0.6998	0.5319	0.3527	0.080*	
H13B	0.7467	0.4370	0.3597	0.080*	
C21A	1.0150 (3)	0.7865 (2)	0.69607 (13)	0.0876 (8)	
H21A	1.0519	0.8202	0.7437	0.105*	
C22A	0.9232 (3)	0.82072 (17)	0.66006 (13)	0.0769 (7)	
C4A	1.2686 (2)	1.05653 (17)	0.65045 (13)	0.0787 (6)	
H4A	1.2271	1.1069	0.6538	0.094*	
C19A	1.0008 (2)	0.65235 (18)	0.58867 (12)	0.0748 (6)	
H19A	1.0264	0.5964	0.5666	0.090*	
C15A	0.7589 (2)	0.71995 (16)	0.47137 (13)	0.0652 (5)	
C27A	1.0143 (2)	0.55366 (17)	0.23487 (13)	0.0702 (6)	
H27A	1.0796	0.5375	0.2601	0.084*	
C1A	1.3969 (2)	0.9090 (2)	0.64031 (16)	0.0923 (8)	
H1A2	1.4413	0.8604	0.6368	0.111*	
C20A	1.0499 (3)	0.7049 (2)	0.66176 (14)	0.0872 (7)	
H20A	1.1086	0.6825	0.6875	0.105*	
C3A	1.3662 (3)	1.0638 (2)	0.70312 (15)	0.0955 (8)	
H3A	1.3899	1.1191	0.7427	0.115*	
C23A	0.8727 (3)	0.9022 (2)	0.68673 (17)	0.1026 (9)	
H23A	0.9018	0.9413	0.7342	0.123*	
C25A	0.7329 (3)	0.8694 (2)	0.57250 (17)	0.0915 (8)	
H25A	0.6703	0.8855	0.5450	0.110*	
C24A	0.7822 (3)	0.9249 (2)	0.64444 (19)	0.1076 (10)	
H24A	0.7521	0.9795	0.6641	0.129*	
C2A	1.4297 (3)	0.9902 (2)	0.69825 (17)	0.1034 (9)	
H2A	1.4951	0.9960	0.7347	0.124*	
Cl2	0.25579 (8)	0.80531 (6)	0.29088 (5)	0.1130 (3)	
N1	−0.42346 (14)	0.50306 (10)	−0.08849 (8)	0.0506 (4)	
H1	−0.4758	0.4639	−0.0658	0.061*	
O2	−0.37887 (13)	0.61898 (9)	0.01806 (7)	0.0578 (3)	
O3	−0.07709 (15)	0.79248 (11)	−0.07932 (8)	0.0808 (5)	
C5	−0.41599 (17)	0.46781 (13)	−0.16319 (9)	0.0460 (4)	
C9	−0.35533 (17)	0.59417 (12)	−0.04758 (10)	0.0481 (4)	
C8	−0.25698 (18)	0.65483 (13)	−0.08473 (10)	0.0506 (4)	
O1	−0.15440 (15)	0.66688 (11)	−0.19654 (8)	0.0807 (5)	
H1B	−0.1102	0.7184	−0.1688	0.121*	
C6	−0.32825 (17)	0.52679 (13)	−0.20136 (9)	0.0493 (4)	
C7	−0.24411 (18)	0.61846 (14)	−0.15949 (10)	0.0538 (5)	
C4	−0.49435 (19)	0.37465 (14)	−0.20066 (10)	0.0562 (5)	
H4	−0.5502	0.3335	−0.1750	0.067*	
C10	−0.1715 (2)	0.75270 (13)	−0.04774 (11)	0.0590 (5)	
N2	−0.24693 (19)	0.96878 (12)	0.03526 (10)	0.0697 (5)	
O4	−0.42244 (19)	0.83028 (12)	0.12114 (9)	0.0954 (5)	
C11	−0.19975 (19)	0.81221 (13)	0.02461 (10)	0.0582 (5)	
H11	−0.2278	0.7670	0.0587	0.070*	
C14	−0.3108 (2)	0.86168 (13)	0.00994 (11)	0.0610 (5)	
C1	−0.3245 (2)	0.49299 (16)	−0.27839 (10)	0.0614 (5)	
H1C	−0.2665	0.5320	−0.3045	0.074*	
C17	−0.5047 (2)	0.77782 (14)	−0.07059 (13)	0.0663 (6)	
C12	−0.0837 (2)	0.90038 (14)	0.06385 (11)	0.0654 (5)	
H12	−0.0290	0.9230	0.0256	0.078*	
C26	0.0001 (2)	0.87571 (14)	0.12084 (11)	0.0619 (5)	
C3	−0.4885 (2)	0.34408 (16)	−0.27551 (11)	0.0635 (5)	
H3	−0.5415	0.2821	−0.3006	0.076*	
C16	−0.5415 (2)	0.76553 (14)	−0.00030 (13)	0.0705 (6)	
C15	−0.4288 (2)	0.81839 (15)	0.05396 (13)	0.0706 (6)	
C2	−0.4049 (2)	0.40392 (17)	−0.31465 (11)	0.0664 (5)	
H2	−0.4041	0.3829	−0.3658	0.080*	
C29	0.1581 (2)	0.83325 (16)	0.22557 (14)	0.0750 (6)	
C18	−0.3755 (2)	0.83578 (14)	−0.06875 (11)	0.0621 (5)	
C13	−0.1477 (2)	0.98141 (15)	0.09448 (12)	0.0738 (6)	
H13C	−0.1853	0.9710	0.1405	0.089*	
H13D	−0.0865	1.0475	0.1032	0.089*	
C27	−0.0457 (2)	0.85014 (16)	0.18572 (12)	0.0702 (6)	
H27	−0.1316	0.8463	0.1942	0.084*	
C28	0.0328 (2)	0.83013 (17)	0.23821 (14)	0.0768 (6)	
H28	0.0005	0.8146	0.2820	0.092*	
C19	−0.3316 (2)	0.85706 (17)	−0.13402 (13)	0.0747 (6)	
H19	−0.2481	0.8976	−0.1347	0.090*	
C22	−0.5882 (2)	0.73713 (17)	−0.13616 (15)	0.0816 (7)	
C30	0.2068 (2)	0.85833 (18)	0.16231 (16)	0.0833 (7)	
H30	0.2926	0.8613	0.1542	0.100*	
C20	−0.4139 (3)	0.8171 (2)	−0.20033 (14)	0.0934 (8)	
H20	−0.3828	0.8313	−0.2447	0.112*	
C31	0.1282 (2)	0.87937 (17)	0.11031 (14)	0.0782 (7)	
H31	0.1620	0.8964	0.0672	0.094*	
C25	−0.6667 (3)	0.70967 (18)	0.00608 (18)	0.0930 (9)	
H25	−0.6943	0.7000	0.0522	0.112*	
C24	−0.7497 (3)	0.6685 (2)	−0.0592 (2)	0.1082 (10)	
H24	−0.8336	0.6309	−0.0557	0.130*	
C23	−0.7139 (3)	0.6808 (2)	−0.1272 (2)	0.1065 (9)	
H23	−0.7733	0.6516	−0.1687	0.128*	
C21	−0.5373 (3)	0.7586 (2)	−0.20198 (16)	0.0985 (8)	
H21	−0.5881	0.7327	−0.2471	0.118*	

### Atomic displacement parameters (Å^2^)

**Table:**

	*U*^11^	*U*^22^	*U*^33^	*U*^12^	*U*^13^	*U*^23^
C32A	0.0926 (18)	0.0802 (17)	0.0974 (18)	0.0097 (14)	0.0261 (15)	0.0334 (14)
C32	0.118 (2)	0.0471 (13)	0.110 (2)	0.0209 (13)	0.0116 (16)	0.0110 (13)
Cl1	0.1231 (5)	0.0841 (4)	0.0700 (4)	0.0476 (4)	0.0330 (3)	0.0220 (3)
O2A	0.0723 (9)	0.0502 (8)	0.0663 (9)	0.0224 (7)	−0.0062 (7)	0.0126 (6)
N1A	0.0671 (10)	0.0449 (9)	0.0631 (10)	0.0162 (8)	−0.0005 (8)	0.0136 (8)
C26A	0.0662 (12)	0.0434 (10)	0.0591 (12)	0.0203 (9)	0.0036 (9)	0.0066 (8)
C9A	0.0557 (11)	0.0468 (11)	0.0559 (11)	0.0126 (9)	0.0083 (9)	0.0178 (9)
C5A	0.0642 (13)	0.0516 (11)	0.0614 (12)	0.0058 (10)	0.0009 (10)	0.0173 (9)
C8A	0.0556 (11)	0.0514 (11)	0.0631 (12)	0.0188 (9)	0.0062 (9)	0.0137 (9)
O1A	0.0820 (11)	0.0842 (12)	0.1344 (15)	0.0466 (10)	−0.0283 (10)	−0.0082 (11)
C12A	0.0648 (12)	0.0447 (10)	0.0625 (12)	0.0194 (9)	0.0018 (9)	0.0110 (9)
C17A	0.0762 (14)	0.0562 (12)	0.0611 (13)	0.0121 (11)	0.0249 (11)	0.0190 (10)
C11A	0.0623 (12)	0.0446 (10)	0.0544 (11)	0.0200 (9)	0.0053 (9)	0.0137 (8)
N2A	0.0746 (11)	0.0531 (10)	0.0670 (11)	0.0132 (8)	0.0123 (9)	0.0195 (8)
O3A	0.0756 (10)	0.0684 (10)	0.0934 (11)	0.0391 (8)	−0.0030 (8)	0.0004 (8)
C1OA	0.0643 (12)	0.0550 (12)	0.0570 (11)	0.0251 (10)	0.0070 (9)	0.0140 (9)
C18A	0.0742 (13)	0.0562 (12)	0.0562 (12)	0.0173 (10)	0.0133 (10)	0.0191 (9)
C29A	0.0816 (15)	0.0504 (11)	0.0605 (12)	0.0258 (10)	0.0139 (11)	0.0070 (9)
C30A	0.0763 (14)	0.0692 (14)	0.0605 (13)	0.0348 (11)	0.0078 (10)	0.0145 (10)
C16A	0.0732 (14)	0.0579 (12)	0.0780 (15)	0.0217 (11)	0.0308 (12)	0.0198 (11)
C6A	0.0605 (13)	0.0599 (13)	0.0774 (14)	0.0123 (10)	−0.0063 (11)	0.0154 (11)
O4A	0.0816 (11)	0.0924 (12)	0.0990 (12)	0.0401 (9)	−0.0063 (10)	0.0212 (10)
C7A	0.0581 (12)	0.0618 (13)	0.0844 (15)	0.0225 (10)	0.0011 (11)	0.0130 (11)
C14A	0.0650 (12)	0.0486 (11)	0.0599 (12)	0.0196 (9)	0.0066 (9)	0.0144 (9)
C31A	0.0691 (13)	0.0711 (14)	0.0627 (13)	0.0348 (11)	0.0124 (10)	0.0151 (10)
C28A	0.0766 (15)	0.0765 (15)	0.0785 (15)	0.0367 (12)	0.0235 (12)	0.0151 (12)
C13A	0.0742 (14)	0.0529 (12)	0.0706 (14)	0.0131 (10)	0.0029 (11)	0.0152 (10)
C21A	0.109 (2)	0.0867 (19)	0.0549 (14)	−0.0004 (16)	0.0100 (13)	0.0230 (13)
C22A	0.0988 (18)	0.0615 (14)	0.0623 (14)	0.0055 (13)	0.0302 (13)	0.0133 (11)
C4A	0.0942 (17)	0.0582 (13)	0.0764 (15)	0.0141 (12)	−0.0101 (13)	0.0100 (11)
C19A	0.0951 (17)	0.0732 (15)	0.0628 (14)	0.0263 (13)	0.0058 (12)	0.0263 (11)
C15A	0.0617 (13)	0.0621 (13)	0.0771 (15)	0.0200 (10)	0.0135 (11)	0.0223 (11)
C27A	0.0682 (14)	0.0758 (15)	0.0742 (15)	0.0342 (12)	0.0066 (11)	0.0143 (11)
C1A	0.0781 (16)	0.0748 (17)	0.116 (2)	0.0172 (13)	−0.0285 (15)	0.0131 (15)
C20A	0.1026 (19)	0.0931 (19)	0.0667 (16)	0.0186 (16)	−0.0004 (13)	0.0323 (14)
C3A	0.115 (2)	0.0696 (16)	0.0849 (18)	0.0073 (15)	−0.0279 (16)	0.0075 (13)
C23A	0.140 (3)	0.0757 (18)	0.0818 (19)	0.0149 (18)	0.0422 (18)	0.0064 (15)
C25A	0.0987 (19)	0.0784 (17)	0.111 (2)	0.0388 (15)	0.0459 (16)	0.0272 (16)
C24A	0.144 (3)	0.0759 (19)	0.108 (2)	0.0412 (19)	0.059 (2)	0.0086 (17)
C2A	0.103 (2)	0.0806 (19)	0.110 (2)	0.0097 (16)	−0.0458 (17)	0.0129 (16)
Cl2	0.0988 (5)	0.0935 (5)	0.1547 (7)	0.0314 (4)	−0.0086 (5)	0.0419 (5)
N1	0.0632 (9)	0.0367 (8)	0.0464 (8)	0.0014 (7)	0.0169 (7)	0.0107 (6)
O2	0.0763 (9)	0.0396 (7)	0.0488 (7)	0.0001 (6)	0.0227 (6)	0.0079 (5)
O3	0.0855 (10)	0.0558 (9)	0.0830 (10)	−0.0116 (8)	0.0347 (8)	0.0110 (7)
C5	0.0508 (10)	0.0422 (9)	0.0467 (10)	0.0144 (8)	0.0083 (8)	0.0110 (8)
C9	0.0592 (11)	0.0365 (9)	0.0474 (10)	0.0079 (8)	0.0133 (8)	0.0122 (8)
C8	0.0608 (11)	0.0374 (9)	0.0518 (10)	0.0059 (8)	0.0170 (9)	0.0140 (8)
O1	0.0915 (11)	0.0686 (9)	0.0683 (9)	−0.0065 (8)	0.0397 (8)	0.0169 (7)
C6	0.0544 (11)	0.0495 (10)	0.0461 (10)	0.0147 (9)	0.0113 (8)	0.0133 (8)
C7	0.0615 (11)	0.0479 (10)	0.0543 (11)	0.0108 (9)	0.0209 (9)	0.0197 (9)
C4	0.0612 (12)	0.0478 (11)	0.0564 (12)	0.0114 (9)	0.0093 (9)	0.0083 (9)
C10	0.0698 (13)	0.0412 (10)	0.0617 (12)	0.0023 (9)	0.0176 (10)	0.0171 (9)
N2	0.0933 (13)	0.0366 (9)	0.0729 (11)	0.0065 (9)	0.0077 (10)	0.0124 (8)
O4	0.1378 (15)	0.0746 (11)	0.0686 (11)	0.0164 (10)	0.0414 (10)	0.0156 (8)
C11	0.0752 (13)	0.0360 (9)	0.0570 (11)	0.0003 (9)	0.0151 (10)	0.0139 (8)
C14	0.0794 (14)	0.0360 (10)	0.0614 (12)	0.0031 (9)	0.0177 (10)	0.0122 (8)
C1	0.0664 (13)	0.0703 (14)	0.0494 (11)	0.0179 (11)	0.0143 (9)	0.0174 (10)
C17	0.0747 (14)	0.0409 (10)	0.0804 (15)	0.0121 (10)	0.0217 (12)	0.0093 (10)
C12	0.0824 (14)	0.0388 (10)	0.0637 (12)	−0.0037 (10)	0.0146 (11)	0.0117 (9)
C26	0.0716 (14)	0.0370 (10)	0.0655 (13)	−0.0014 (9)	0.0144 (10)	0.0051 (9)
C3	0.0696 (13)	0.0581 (12)	0.0567 (12)	0.0169 (10)	0.0027 (10)	−0.0004 (10)
C16	0.0788 (15)	0.0378 (10)	0.0905 (16)	0.0100 (10)	0.0313 (13)	0.0078 (10)
C15	0.1028 (17)	0.0433 (11)	0.0695 (14)	0.0213 (11)	0.0354 (13)	0.0149 (10)
C2	0.0727 (14)	0.0775 (15)	0.0465 (11)	0.0233 (12)	0.0063 (10)	0.0039 (10)
C29	0.0734 (15)	0.0498 (12)	0.0979 (18)	0.0138 (11)	0.0048 (13)	0.0119 (12)
C18	0.0735 (14)	0.0428 (10)	0.0664 (13)	0.0075 (10)	0.0147 (10)	0.0135 (9)
C13	0.1017 (17)	0.0371 (10)	0.0706 (14)	0.0009 (11)	0.0072 (13)	0.0083 (10)
C27	0.0717 (14)	0.0623 (13)	0.0808 (15)	0.0152 (11)	0.0207 (12)	0.0273 (11)
C28	0.0837 (16)	0.0668 (14)	0.0847 (16)	0.0172 (12)	0.0163 (13)	0.0314 (12)
C19	0.0835 (15)	0.0653 (14)	0.0730 (15)	0.0081 (12)	0.0142 (12)	0.0255 (11)
C22	0.0813 (17)	0.0539 (13)	0.0967 (19)	0.0099 (12)	0.0047 (14)	−0.0015 (13)
C30	0.0667 (15)	0.0714 (16)	0.0969 (19)	0.0095 (12)	0.0137 (14)	−0.0047 (14)
C20	0.111 (2)	0.096 (2)	0.0705 (17)	0.0189 (17)	0.0093 (15)	0.0260 (14)
C31	0.0791 (16)	0.0631 (14)	0.0725 (15)	−0.0052 (12)	0.0193 (13)	−0.0002 (11)
C25	0.0989 (19)	0.0519 (13)	0.127 (2)	0.0150 (13)	0.0582 (18)	0.0150 (14)
C24	0.0811 (19)	0.0586 (16)	0.159 (3)	−0.0052 (13)	0.029 (2)	−0.0060 (18)
C23	0.0848 (19)	0.0738 (18)	0.137 (3)	0.0020 (15)	0.0116 (18)	−0.0076 (17)
C21	0.106 (2)	0.094 (2)	0.0838 (19)	0.0171 (17)	−0.0022 (16)	0.0084 (15)

### Geometric parameters (Å, °)

**Table:**

C32A—N2A	1.455 (3)	C25A—C24A	1.404 (4)
C32A—H32A	0.9600	C25A—H25A	0.9300
C32A—H32B	0.9600	C24A—H24A	0.9300
C32A—H32C	0.9600	C2A—H2A	0.9300
C32—N2	1.451 (3)	Cl2—C29	1.738 (3)
C32—H32D	0.9600	N1—C9	1.359 (2)
C32—H32E	0.9600	N1—C5	1.379 (2)
C32—H32F	0.9600	N1—H1	0.8600
Cl1—C29A	1.737 (2)	O2—C9	1.2431 (19)
O2A—C9A	1.242 (2)	O3—C10	1.244 (2)
N1A—C9A	1.363 (2)	C5—C6	1.392 (2)
N1A—C5A	1.372 (2)	C5—C4	1.393 (2)
N1A—H1A	0.8600	C9—C8	1.456 (2)
C26A—C31A	1.381 (3)	C8—C7	1.395 (2)
C26A—C27A	1.385 (3)	C8—C10	1.463 (3)
C26A—C12A	1.506 (3)	O1—C7	1.319 (2)
C9A—C8A	1.454 (3)	O1—H1B	0.8200
C5A—C6A	1.391 (3)	C6—C1	1.409 (3)
C5A—C4A	1.397 (3)	C6—C7	1.426 (3)
C8A—C7A	1.391 (3)	C4—C3	1.368 (3)
C8A—C1OA	1.464 (3)	C4—H4	0.9300
O1A—C7A	1.325 (2)	C10—C11	1.515 (3)
O1A—H1'	0.8200	N2—C13	1.448 (3)
C12A—C13A	1.516 (3)	N2—C14	1.462 (2)
C12A—C11A	1.553 (3)	O4—C15	1.210 (3)
C12A—H12A	0.9800	C11—C12	1.547 (3)
C17A—C18A	1.403 (3)	C11—C14	1.576 (3)
C17A—C22A	1.404 (3)	C11—H11	0.9800
C17A—C16A	1.400 (3)	C14—C18	1.516 (3)
C11A—C1OA	1.509 (3)	C14—C15	1.578 (3)
C11A—C14A	1.576 (3)	C1—C2	1.355 (3)
C11A—H11A	0.9800	C1—H1C	0.9300
N2A—C13A	1.453 (3)	C17—C16	1.394 (3)
N2A—C14A	1.467 (2)	C17—C22	1.403 (3)
O3A—C1OA	1.243 (2)	C17—C18	1.408 (3)
C18A—C19A	1.366 (3)	C12—C26	1.509 (3)
C18A—C14A	1.513 (3)	C12—C13	1.516 (3)
C29A—C30A	1.366 (3)	C12—H12	0.9800
C29A—C28A	1.358 (3)	C26—C27	1.382 (3)
C30A—C31A	1.381 (3)	C26—C31	1.385 (3)
C30A—H30A	0.9300	C3—C2	1.386 (3)
C16A—C25A	1.378 (3)	C3—H3	0.9300
C16A—C15A	1.474 (3)	C16—C25	1.390 (3)
C6A—C1A	1.402 (3)	C16—C15	1.472 (3)
C6A—C7A	1.431 (3)	C2—H2	0.9300
O4A—C15A	1.205 (2)	C29—C28	1.365 (3)
C14A—C15A	1.567 (3)	C29—C30	1.363 (3)
C31A—H31A	0.9300	C18—C19	1.363 (3)
C28A—C27A	1.377 (3)	C13—H13C	0.9700
C28A—H28A	0.9300	C13—H13D	0.9700
C13A—H13A	0.9700	C27—C28	1.379 (3)
C13A—H13B	0.9700	C27—H27	0.9300
C21A—C20A	1.356 (4)	C28—H28	0.9300
C21A—C22A	1.407 (4)	C19—C20	1.407 (3)
C21A—H21A	0.9300	C19—H19	0.9300
C22A—C23A	1.412 (4)	C22—C21	1.399 (4)
C4A—C3A	1.372 (3)	C22—C23	1.409 (4)
C4A—H4A	0.9300	C30—C31	1.381 (3)
C19A—C20A	1.410 (3)	C30—H30	0.9300
C19A—H19A	0.9300	C20—C21	1.360 (4)
C27A—H27A	0.9300	C20—H20	0.9300
C1A—C2A	1.364 (4)	C31—H31	0.9300
C1A—H1A2	0.9300	C25—C24	1.397 (4)
C20A—H20A	0.9300	C25—H25	0.9300
C3A—C2A	1.380 (4)	C24—C23	1.350 (4)
C3A—H3A	0.9300	C24—H24	0.9300
C23A—C24A	1.366 (4)	C23—H23	0.9300
C23A—H23A	0.9300	C21—H21	0.9300
			
N2A—C32A—H32A	109.5	C23A—C24A—C25A	122.8 (3)
N2A—C32A—H32B	109.5	C23A—C24A—H24A	118.6
H32A—C32A—H32B	109.5	C25A—C24A—H24A	118.6
N2A—C32A—H32C	109.5	C1A—C2A—C3A	120.3 (2)
H32A—C32A—H32C	109.5	C1A—C2A—H2A	119.8
H32B—C32A—H32C	109.5	C3A—C2A—H2A	119.8
N2—C32—H32D	109.5	C9—N1—C5	125.77 (14)
N2—C32—H32E	109.5	C9—N1—H1	117.1
H32D—C32—H32E	109.5	C5—N1—H1	117.1
N2—C32—H32F	109.5	N1—C5—C6	119.05 (16)
H32D—C32—H32F	109.5	N1—C5—C4	121.15 (15)
H32E—C32—H32F	109.5	C6—C5—C4	119.81 (16)
C9A—N1A—C5A	125.23 (16)	O2—C9—N1	118.77 (15)
C9A—N1A—H1A	117.4	O2—C9—C8	124.93 (16)
C5A—N1A—H1A	117.4	N1—C9—C8	116.28 (15)
C31A—C26A—C27A	117.35 (19)	C7—C8—C9	118.54 (16)
C31A—C26A—C12A	122.17 (17)	C7—C8—C10	118.20 (15)
C27A—C26A—C12A	120.46 (18)	C9—C8—C10	123.25 (16)
O2A—C9A—N1A	119.11 (16)	C7—O1—H1B	109.5
O2A—C9A—C8A	124.22 (18)	C5—C6—C1	119.16 (17)
N1A—C9A—C8A	116.67 (17)	C5—C6—C7	117.66 (16)
N1A—C5A—C6A	119.46 (19)	C1—C6—C7	123.16 (16)
N1A—C5A—C4A	120.48 (19)	O1—C7—C8	122.43 (17)
C6A—C5A—C4A	120.1 (2)	O1—C7—C6	115.39 (16)
C7A—C8A—C9A	118.57 (18)	C8—C7—C6	122.18 (15)
C7A—C8A—C1OA	118.10 (17)	C3—C4—C5	119.52 (18)
C9A—C8A—C1OA	122.87 (17)	C3—C4—H4	120.2
C7A—O1A—H1'	109.5	C5—C4—H4	120.2
C26A—C12A—C13A	117.09 (17)	O3—C10—C8	119.10 (17)
C26A—C12A—C11A	113.32 (15)	O3—C10—C11	118.16 (16)
C13A—C12A—C11A	103.07 (15)	C8—C10—C11	122.50 (16)
C26A—C12A—H12A	107.6	C13—N2—C32	115.03 (18)
C13A—C12A—H12A	107.6	C13—N2—C14	108.30 (16)
C11A—C12A—H12A	107.6	C32—N2—C14	116.67 (19)
C18A—C17A—C22A	123.3 (2)	C10—C11—C12	114.20 (16)
C18A—C17A—C16A	113.06 (19)	C10—C11—C14	110.36 (16)
C22A—C17A—C16A	123.6 (2)	C12—C11—C14	105.11 (15)
C1OA—C11A—C12A	113.86 (15)	C10—C11—H11	109.0
C1OA—C11A—C14A	111.75 (15)	C12—C11—H11	109.0
C12A—C11A—C14A	105.22 (15)	C14—C11—H11	109.0
C1OA—C11A—H11A	108.6	N2—C14—C18	112.58 (16)
C12A—C11A—H11A	108.6	N2—C14—C11	102.88 (16)
C14A—C11A—H11A	108.6	C18—C14—C11	117.16 (16)
C13A—N2A—C32A	114.45 (18)	N2—C14—C15	115.27 (16)
C13A—N2A—C14A	107.68 (15)	C18—C14—C15	101.09 (17)
C32A—N2A—C14A	114.88 (17)	C11—C14—C15	108.35 (15)
O3A—C1OA—C8A	119.25 (18)	C2—C1—C6	120.30 (18)
O3A—C1OA—C11A	118.60 (18)	C2—C1—H1C	119.8
C8A—C1OA—C11A	121.74 (16)	C6—C1—H1C	119.8
C19A—C18A—C17A	118.7 (2)	C16—C17—C22	123.4 (2)
C19A—C18A—C14A	131.97 (19)	C16—C17—C18	113.0 (2)
C17A—C18A—C14A	109.37 (18)	C22—C17—C18	123.7 (2)
C30A—C29A—C28A	120.9 (2)	C26—C12—C13	114.77 (17)
C30A—C29A—Cl1	118.98 (17)	C26—C12—C11	114.95 (15)
C28A—C29A—Cl1	120.12 (16)	C13—C12—C11	102.89 (18)
C29A—C30A—C31A	119.4 (2)	C26—C12—H12	107.9
C29A—C30A—H30A	120.3	C13—C12—H12	107.9
C31A—C30A—H30A	120.3	C11—C12—H12	107.9
C25A—C16A—C17A	119.9 (2)	C27—C26—C31	117.1 (2)
C25A—C16A—C15A	132.5 (2)	C27—C26—C12	122.20 (19)
C17A—C16A—C15A	107.48 (18)	C31—C26—C12	120.7 (2)
C5A—C6A—C1A	119.2 (2)	C4—C3—C2	121.1 (2)
C5A—C6A—C7A	117.73 (18)	C4—C3—H3	119.4
C1A—C6A—C7A	123.0 (2)	C2—C3—H3	119.4
O1A—C7A—C8A	121.9 (2)	C25—C16—C17	119.1 (2)
O1A—C7A—C6A	116.07 (19)	C25—C16—C15	133.4 (2)
C8A—C7A—C6A	121.84 (18)	C17—C16—C15	107.54 (18)
N2A—C14A—C18A	111.66 (15)	O4—C15—C16	128.3 (2)
N2A—C14A—C15A	114.73 (17)	O4—C15—C14	123.4 (2)
C18A—C14A—C15A	102.04 (16)	C16—C15—C14	108.26 (18)
N2A—C14A—C11A	102.73 (14)	C1—C2—C3	120.00 (19)
C18A—C14A—C11A	117.97 (16)	C1—C2—H2	120.0
C15A—C14A—C11A	108.19 (14)	C3—C2—H2	120.0
C30A—C31A—C26A	121.32 (19)	C28—C29—C30	120.4 (2)
C30A—C31A—H31A	119.3	C28—C29—Cl2	119.6 (2)
C26A—C31A—H31A	119.3	C30—C29—Cl2	120.1 (2)
C29A—C28A—C27A	119.4 (2)	C19—C18—C17	117.8 (2)
C29A—C28A—H28A	120.3	C19—C18—C14	132.4 (2)
C27A—C28A—H28A	120.3	C17—C18—C14	109.82 (17)
N2A—C13A—C12A	102.47 (16)	N2—C13—C12	102.28 (17)
N2A—C13A—H13A	111.3	N2—C13—H13C	111.3
C12A—C13A—H13A	111.3	C12—C13—H13C	111.3
N2A—C13A—H13B	111.3	N2—C13—H13D	111.3
C12A—C13A—H13B	111.3	C12—C13—H13D	111.3
H13A—C13A—H13B	109.2	H13C—C13—H13D	109.2
C20A—C21A—C22A	120.5 (2)	C26—C27—C28	121.6 (2)
C20A—C21A—H21A	119.8	C26—C27—H27	119.2
C22A—C21A—H21A	119.8	C28—C27—H27	119.2
C17A—C22A—C23A	114.9 (3)	C29—C28—C27	119.7 (2)
C17A—C22A—C21A	116.2 (2)	C29—C28—H28	120.2
C23A—C22A—C21A	128.9 (3)	C27—C28—H28	120.2
C3A—C4A—C5A	119.3 (2)	C18—C19—C20	119.3 (2)
C3A—C4A—H4A	120.3	C18—C19—H19	120.3
C5A—C4A—H4A	120.3	C20—C19—H19	120.3
C18A—C19A—C20A	118.7 (2)	C21—C22—C17	116.3 (2)
C18A—C19A—H19A	120.7	C21—C22—C23	128.1 (3)
C20A—C19A—H19A	120.7	C17—C22—C23	115.6 (3)
O4A—C15A—C16A	127.9 (2)	C29—C30—C31	119.7 (2)
O4A—C15A—C14A	124.3 (2)	C29—C30—H30	120.2
C16A—C15A—C14A	107.87 (18)	C31—C30—H30	120.2
C28A—C27A—C26A	121.6 (2)	C21—C20—C19	122.5 (2)
C28A—C27A—H27A	119.2	C21—C20—H20	118.8
C26A—C27A—H27A	119.2	C19—C20—H20	118.8
C2A—C1A—C6A	120.2 (2)	C30—C31—C26	121.5 (2)
C2A—C1A—H1A2	119.9	C30—C31—H31	119.3
C6A—C1A—H1A2	119.9	C26—C31—H31	119.3
C21A—C20A—C19A	122.6 (2)	C24—C25—C16	117.6 (3)
C21A—C20A—H20A	118.7	C24—C25—H25	121.2
C19A—C20A—H20A	118.7	C16—C25—H25	121.2
C4A—C3A—C2A	120.9 (2)	C23—C24—C25	123.2 (3)
C4A—C3A—H3A	119.5	C23—C24—H24	118.4
C2A—C3A—H3A	119.5	C25—C24—H24	118.4
C24A—C23A—C22A	121.5 (3)	C24—C23—C22	121.1 (3)
C24A—C23A—H23A	119.2	C24—C23—H23	119.5
C22A—C23A—H23A	119.2	C22—C23—H23	119.5
C16A—C25A—C24A	117.3 (3)	C20—C21—C22	120.4 (3)
C16A—C25A—H25A	121.4	C20—C21—H21	119.8
C24A—C25A—H25A	121.4	C22—C21—H21	119.8
			
C5A—N1A—C9A—O2A	178.65 (17)	C9—N1—C5—C6	2.8 (3)
C5A—N1A—C9A—C8A	−1.5 (3)	C9—N1—C5—C4	−177.24 (17)
C9A—N1A—C5A—C6A	5.3 (3)	C5—N1—C9—O2	174.73 (16)
C9A—N1A—C5A—C4A	−173.83 (19)	C5—N1—C9—C8	−6.5 (3)
O2A—C9A—C8A—C7A	174.88 (18)	O2—C9—C8—C7	−178.09 (17)
N1A—C9A—C8A—C7A	−5.0 (3)	N1—C9—C8—C7	3.3 (3)
O2A—C9A—C8A—C1OA	−13.1 (3)	O2—C9—C8—C10	1.3 (3)
N1A—C9A—C8A—C1OA	167.00 (17)	N1—C9—C8—C10	−177.30 (17)
C31A—C26A—C12A—C13A	39.2 (3)	N1—C5—C6—C1	−177.17 (16)
C27A—C26A—C12A—C13A	−142.7 (2)	C4—C5—C6—C1	2.9 (3)
C31A—C26A—C12A—C11A	−80.6 (2)	N1—C5—C6—C7	4.1 (2)
C27A—C26A—C12A—C11A	97.5 (2)	C4—C5—C6—C7	−175.84 (16)
C26A—C12A—C11A—C1OA	−91.64 (19)	C9—C8—C7—O1	−177.44 (17)
C13A—C12A—C11A—C1OA	140.83 (16)	C10—C8—C7—O1	3.1 (3)
C26A—C12A—C11A—C14A	145.66 (16)	C9—C8—C7—C6	3.3 (3)
C13A—C12A—C11A—C14A	18.14 (18)	C10—C8—C7—C6	−176.12 (17)
C7A—C8A—C1OA—O3A	−18.4 (3)	C5—C6—C7—O1	173.65 (16)
C9A—C8A—C1OA—O3A	169.59 (18)	C1—C6—C7—O1	−5.0 (3)
C7A—C8A—C1OA—C11A	154.14 (18)	C5—C6—C7—C8	−7.1 (3)
C9A—C8A—C1OA—C11A	−17.9 (3)	C1—C6—C7—C8	174.24 (18)
C12A—C11A—C1OA—O3A	−14.0 (2)	N1—C5—C4—C3	177.04 (17)
C14A—C11A—C1OA—O3A	105.0 (2)	C6—C5—C4—C3	−3.0 (3)
C12A—C11A—C1OA—C8A	173.43 (16)	C7—C8—C10—O3	−11.8 (3)
C14A—C11A—C1OA—C8A	−67.5 (2)	C9—C8—C10—O3	168.74 (18)
C22A—C17A—C18A—C19A	3.7 (3)	C7—C8—C10—C11	162.55 (18)
C16A—C17A—C18A—C19A	−176.78 (18)	C9—C8—C10—C11	−16.9 (3)
C22A—C17A—C18A—C14A	−175.55 (18)	O3—C10—C11—C12	−19.2 (3)
C16A—C17A—C18A—C14A	4.0 (2)	C8—C10—C11—C12	166.32 (17)
C28A—C29A—C30A—C31A	1.4 (3)	O3—C10—C11—C14	98.9 (2)
Cl1—C29A—C30A—C31A	−177.19 (17)	C8—C10—C11—C14	−75.5 (2)
C18A—C17A—C16A—C25A	−179.23 (19)	C13—N2—C14—C18	−157.09 (18)
C22A—C17A—C16A—C25A	0.3 (3)	C32—N2—C14—C18	71.2 (2)
C18A—C17A—C16A—C15A	−1.8 (2)	C13—N2—C14—C11	−30.1 (2)
C22A—C17A—C16A—C15A	177.77 (19)	C32—N2—C14—C11	−161.73 (18)
N1A—C5A—C6A—C1A	−178.5 (2)	C13—N2—C14—C15	87.7 (2)
C4A—C5A—C6A—C1A	0.6 (3)	C32—N2—C14—C15	−44.0 (3)
N1A—C5A—C6A—C7A	−2.5 (3)	C10—C11—C14—N2	−119.19 (16)
C4A—C5A—C6A—C7A	176.7 (2)	C12—C11—C14—N2	4.39 (18)
C9A—C8A—C7A—O1A	−176.95 (19)	C10—C11—C14—C18	4.9 (2)
C1OA—C8A—C7A—O1A	10.7 (3)	C12—C11—C14—C18	128.44 (16)
C9A—C8A—C7A—C6A	7.8 (3)	C10—C11—C14—C15	118.31 (17)
C1OA—C8A—C7A—C6A	−164.64 (19)	C12—C11—C14—C15	−118.11 (17)
C5A—C6A—C7A—O1A	−179.6 (2)	C5—C6—C1—C2	−0.3 (3)
C1A—C6A—C7A—O1A	−3.6 (3)	C7—C6—C1—C2	178.35 (19)
C5A—C6A—C7A—C8A	−4.0 (3)	C10—C11—C12—C26	−92.7 (2)
C1A—C6A—C7A—C8A	171.9 (2)	C14—C11—C12—C26	146.23 (17)
C13A—N2A—C14A—C18A	−159.82 (16)	C10—C11—C12—C13	141.83 (17)
C32A—N2A—C14A—C18A	71.4 (2)	C14—C11—C12—C13	20.75 (19)
C13A—N2A—C14A—C15A	84.7 (2)	C13—C12—C26—C27	53.7 (2)
C32A—N2A—C14A—C15A	−44.1 (2)	C11—C12—C26—C27	−65.4 (2)
C13A—N2A—C14A—C11A	−32.44 (19)	C13—C12—C26—C31	−125.4 (2)
C32A—N2A—C14A—C11A	−161.25 (17)	C11—C12—C26—C31	115.6 (2)
C19A—C18A—C14A—N2A	53.7 (3)	C5—C4—C3—C2	0.6 (3)
C17A—C18A—C14A—N2A	−127.23 (17)	C22—C17—C16—C25	0.5 (3)
C19A—C18A—C14A—C15A	176.7 (2)	C18—C17—C16—C25	−179.89 (19)
C17A—C18A—C14A—C15A	−4.2 (2)	C22—C17—C16—C15	179.9 (2)
C19A—C18A—C14A—C11A	−65.0 (3)	C18—C17—C16—C15	−0.5 (2)
C17A—C18A—C14A—C11A	114.12 (18)	C25—C16—C15—O4	−5.0 (4)
C1OA—C11A—C14A—N2A	−116.54 (16)	C17—C16—C15—O4	175.7 (2)
C12A—C11A—C14A—N2A	7.50 (18)	C25—C16—C15—C14	176.1 (2)
C1OA—C11A—C14A—C18A	6.7 (2)	C17—C16—C15—C14	−3.2 (2)
C12A—C11A—C14A—C18A	130.75 (17)	N2—C14—C15—O4	−52.1 (3)
C1OA—C11A—C14A—C15A	121.74 (17)	C18—C14—C15—O4	−173.7 (2)
C12A—C11A—C14A—C15A	−114.22 (17)	C11—C14—C15—O4	62.6 (2)
C29A—C30A—C31A—C26A	−0.8 (3)	N2—C14—C15—C16	126.98 (19)
C27A—C26A—C31A—C30A	−0.7 (3)	C18—C14—C15—C16	5.3 (2)
C12A—C26A—C31A—C30A	177.51 (19)	C11—C14—C15—C16	−118.42 (18)
C30A—C29A—C28A—C27A	−0.5 (3)	C6—C1—C2—C3	−2.1 (3)
Cl1—C29A—C28A—C27A	178.10 (18)	C4—C3—C2—C1	2.0 (3)
C32A—N2A—C13A—C12A	174.14 (17)	C16—C17—C18—C19	−176.49 (19)
C14A—N2A—C13A—C12A	45.08 (19)	C22—C17—C18—C19	3.1 (3)
C26A—C12A—C13A—N2A	−162.62 (15)	C16—C17—C18—C14	4.2 (2)
C11A—C12A—C13A—N2A	−37.51 (18)	C22—C17—C18—C14	−176.22 (19)
C18A—C17A—C22A—C23A	178.5 (2)	N2—C14—C18—C19	51.6 (3)
C16A—C17A—C22A—C23A	−1.0 (3)	C11—C14—C18—C19	−67.3 (3)
C18A—C17A—C22A—C21A	−1.5 (3)	C15—C14—C18—C19	175.2 (2)
C16A—C17A—C22A—C21A	179.03 (19)	N2—C14—C18—C17	−129.17 (18)
C20A—C21A—C22A—C17A	−1.4 (3)	C11—C14—C18—C17	111.84 (19)
C20A—C21A—C22A—C23A	178.6 (2)	C15—C14—C18—C17	−5.6 (2)
N1A—C5A—C4A—C3A	177.7 (2)	C32—N2—C13—C12	176.79 (17)
C6A—C5A—C4A—C3A	−1.4 (3)	C14—N2—C13—C12	44.2 (2)
C17A—C18A—C19A—C20A	−2.9 (3)	C26—C12—C13—N2	−164.24 (16)
C14A—C18A—C19A—C20A	176.1 (2)	C11—C12—C13—N2	−38.63 (18)
C25A—C16A—C15A—O4A	−3.7 (4)	C31—C26—C27—C28	0.7 (3)
C17A—C16A—C15A—O4A	179.3 (2)	C12—C26—C27—C28	−178.40 (19)
C25A—C16A—C15A—C14A	175.9 (2)	C30—C29—C28—C27	1.6 (3)
C17A—C16A—C15A—C14A	−1.1 (2)	Cl2—C29—C28—C27	−178.59 (17)
N2A—C14A—C15A—O4A	−56.3 (3)	C26—C27—C28—C29	−1.5 (3)
C18A—C14A—C15A—O4A	−177.2 (2)	C17—C18—C19—C20	−2.8 (3)
C11A—C14A—C15A—O4A	57.7 (3)	C14—C18—C19—C20	176.3 (2)
N2A—C14A—C15A—C16A	124.08 (18)	C16—C17—C22—C21	178.3 (2)
C18A—C14A—C15A—C16A	3.2 (2)	C18—C17—C22—C21	−1.2 (3)
C11A—C14A—C15A—C16A	−121.92 (17)	C16—C17—C22—C23	−0.7 (3)
C29A—C28A—C27A—C26A	−1.1 (3)	C18—C17—C22—C23	179.7 (2)
C31A—C26A—C27A—C28A	1.6 (3)	C28—C29—C30—C31	−0.9 (3)
C12A—C26A—C27A—C28A	−176.6 (2)	Cl2—C29—C30—C31	179.30 (17)
C5A—C6A—C1A—C2A	0.8 (4)	C18—C19—C20—C21	0.9 (4)
C7A—C6A—C1A—C2A	−175.1 (3)	C29—C30—C31—C26	0.1 (3)
C22A—C21A—C20A—C19A	2.1 (4)	C27—C26—C31—C30	0.0 (3)
C18A—C19A—C20A—C21A	0.1 (4)	C12—C26—C31—C30	179.13 (19)
C5A—C4A—C3A—C2A	0.9 (4)	C17—C16—C25—C24	−0.1 (3)
C17A—C22A—C23A—C24A	0.6 (4)	C15—C16—C25—C24	−179.3 (2)
C21A—C22A—C23A—C24A	−179.4 (3)	C16—C25—C24—C23	−0.2 (4)
C17A—C16A—C25A—C24A	0.7 (3)	C25—C24—C23—C22	−0.1 (5)
C15A—C16A—C25A—C24A	−176.0 (2)	C21—C22—C23—C24	−178.4 (3)
C22A—C23A—C24A—C25A	0.4 (4)	C17—C22—C23—C24	0.5 (4)
C16A—C25A—C24A—C23A	−1.1 (4)	C19—C20—C21—C22	1.1 (5)
C6A—C1A—C2A—C3A	−1.4 (5)	C17—C22—C21—C20	−0.9 (4)
C4A—C3A—C2A—C1A	0.5 (5)	C23—C22—C21—C20	178.0 (3)

### Hydrogen-bond geometry (Å, °)

**Table:**

*D*—H···*A*	*D*—H	H···*A*	*D*···*A*	*D*—H···*A*
N1—H1···O2^i^	0.86	2.03	2.879 (2)	171
O1—H1B···O3	0.82	1.74	2.468 (2)	147
N1A—H1A···O2A^ii^	0.86	2.01	2.865 (2)	174
O1A—H1'···O3A	0.82	1.75	2.477 (2)	148
C3A—H3A···O4^iii^	0.93	2.45	3.245 (3)	143
C13—H13D···O3^iv^	0.97	2.60	3.530 (3)	161
C30A—H30A···O2^v^	0.93	2.54	3.413 (2)	157

Symmetry codes: (i) −*x*−1, −*y*+1, −*z*;  (ii) −*x*+2, −*y*+2, −*z*+1; (iii) −*x*+1, −*y*+2, −*z*+1; (iv) −*x*, −*y*+2, −*z*; (v) *x*+1, *y*, *z*.
